# HCV Interplay With Mir34a: Implications in Hepatocellular Carcinoma

**DOI:** 10.3389/fonc.2021.803278

**Published:** 2022-01-19

**Authors:** Ester Badami, Claudia Carcione, Cinzia Maria Chinnici, Rosaria Tinnirello, Pier Giulio Conaldi, Gioacchin Iannolo

**Affiliations:** ^1^ Department of Research, Istituto di Ricovero e Cura a Carattere Scientifico Istituto Mediterraneo per i Trapianti e Terapie ad Alta Specializzazione (IRCCS ISMETT), Palermo, Italy; ^2^ Regenerative Medicine and Immunotherapy Area, Fondazione Ri.MED, Palermo, Italy; ^3^ Neuroscience Unit, Consiglio Nazionale delle Ricerche (CNR), Institute of Biomedicine and Molecular Immunology, Palermo, Italy

**Keywords:** HCC, liver, HCV, miR34, DAA, EV, transplantation

## Abstract

Since its identification, HCV has been considered one of the main causes of hepatitis and liver cancer. Currently, the molecular mechanisms of HCC development induced by HCV infection have not been sufficiently clarified. The recent discovery of novel treatments that inhibit HCV replication gave rise to new questions concerning HCC mechanisms. In particular, the HCV eradication mediated by new direct-acting antiviral (DAAs) drugs does not exclude the possibility of *de novo* HCC development; this finding opened more questions on the interplay between liver cells and the virus. Different groups have investigated the pathways leading to cancer recurrence in patients treated with DAAs. For this reason, we tried to gain molecular insights into the changes induced by HCV infection in the target liver cells. In particular, we observed an increase in microRNA34a (miR34a) expression following HCV infection of HCC cell line Huh7.5. In addition, Huh7.5 treated with extracellular vesicles (EVs) from the previously HCV-infected Huh7.5 underwent apoptosis. Since miR34 expression was increased in Huh7.5 EVs, we hypothesized a paracrine mechanism of viral infection mediated by miR34a cargo of EVs. The balance between viral infection and cell transformation may raise some questions on the possible use of antiviral drugs in association with antineoplastic treatment.

## Introduction

Liver cancer is the fourth cause of cancer death worldwide (1), among which hepatocellular carcinoma (HCC) accounts for 75%–85%. Chronic infection caused by hepatitis C virus (HCV) is one of the most common HCC risk factors (1). The HCV genome was originally identified in 1989 (2). The virus belongs to the Flaviviridae family and primarily infects hepatocytes (3). After infection, the positive single-stranded (+) RNA viral genome is translated *via* cellular ribosomal apparatus and copied to a negative strand (–), generating a replicative intermediate (RI). Apart from this, there is no well-defined connection for HCV infection and HCC induction, although various mechanisms have been evaluated (4–6). At present, direct-acting antivirals (DAAs) represent a long-expected solution for HCV treatment (7, 8), and several studies demonstrated the benefit of DAA regimen before HCC diagnosis, with an increased median survival up to 5 years (9). Nevertheless, interferon (IFN)-free DAA treatment is well tolerated and was associated with improved survival (10); recent reports showed that DAA treatment does not completely avoid the occurrence of HCC (11–13), raising a great debate on the consequence of this treatment and the need for screening after viral eradication (14, 15).

This requirement is strictly necessary in liver transplantation, where the use of DAA therapy opened new chances for HCV-positive recipients (16) and for HCV-positive donors into HCV-negative recipients (17, 18).

Zika virus (ZIKV) is another member of the Flaviviridae family: ZIKV infection is usually asymptomatic but during pregnancy induces neural developmental malformation, such as microcephaly. Defects of fetal neurogenesis have been ascribed to the effect of ZIKV on neural stem cells (NSCs), which are specifically targeted, thus resulting in central nervous system (SNC) abnormality (19). An additional target of ZIKV is glioblastoma stem cells (GSCs), which are the transformed counterpart of NSCs. It was observed that the virus displays an oncolytic effect on glioblastoma (20, 21). To dissect the oncolytic mechanism of ZIKV, we previously performed a next-generation sequencing (NGS) analysis of GSCs after viral infection. Our results suggested that ZIKV infection induces a boost in cellular expression of miR34c (21). In addition, the apoptotic effect observed in GSCs following ZIKV infection was associated with the induction of miR34c expression (21). MiR34c belongs to the miR34 family, which includes miR34a and miR34b. Both miRNAs are involved at various levels with senescence (22), stemness (23) and neoplastic progression (24).

To assess whether HCV can act similarly to ZIKV and exert a cytostatic/cytotoxic effect in target cells, we evaluated an HCV-induced miR34 expression in Huh7.5 cell lines. We observed a clear increase of miR34 expression following HCV infection. Moreover, in our work we demonstrated that the effect could act not only on the infected cells but also in a paracrine manner mediated by EVs.

## Results

In order to evaluate whether HCV infection can cause an increase of miR34 expression in target cells, as observed for ZIKV, and induce a cytostatic/cytotoxic effect, we addressed molecular changes in Huh7.5 before and after HCV infection. This cell line was selected as the gold-standard model to study HCV infection because it involves the most permissive cells for viral infection and sustained productive replication (25, 26). First, we performed a TaqMan assay to accurately quantify miR34 expression.

**Figure 1:**
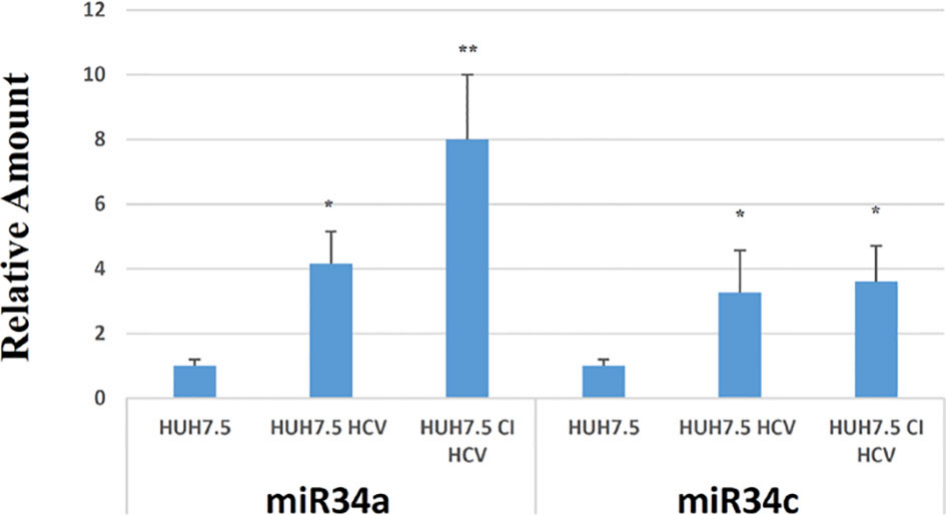
RT-PCR analysis for miR34a and miR34c in empty Huh7.5 (control), HCV infected and Huh7.5 CI. Values are expressed as fold change vs. the control. Data are representative of experiments performed in triplicate on three different samples (p value * ≤0.05, ** ≤0.005).

For this purpose, we infected Huh7.5 cells with recombinant HCV replicons and studied miR34 expression (acute infection). For comparison, we used a Huh7.5-derived cell line that we generated by long-term culture, stably transfected with HCV DNA (Huh7.5-CI-HCV) mimicking a chronic infection in an *in vitro* model. This analysis revealed a significant increase in miR34a and miR34c expression in both cell lines infected with HCV, while the miR34b level was almost undetectable (data not shown). In particular, we observed a 4-fold and 8-fold increase in miR34a expression in Huh7.5 HCV and Huh7.5-CI-HCV vs. non-infected Huh7.5 control, respectively (**Figure 1**). Quantification of miR34-c resulted in an almost 4-fold increase in both acute and chronic cell lines compared to non-infected Huh7.5 cells (**Figure 1**).

**Figure 2:**
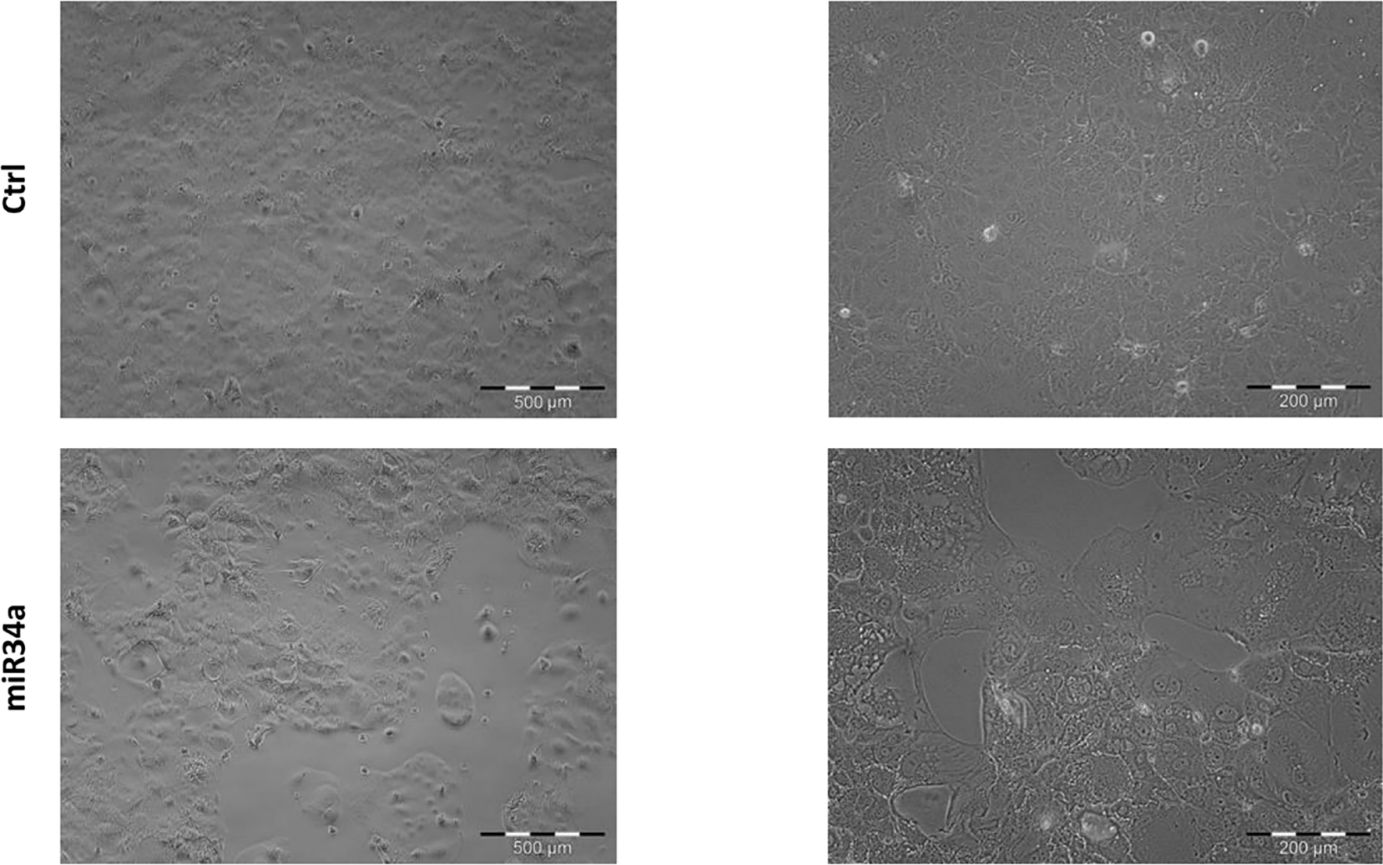
Morphological evaluation of Huh7.5 control and overexpressing miR34a in bright-field microscopy. The cells’ cell morphology after infection appears clearly modified, and the cellular density is lower than control cells.

**Figure 3:**
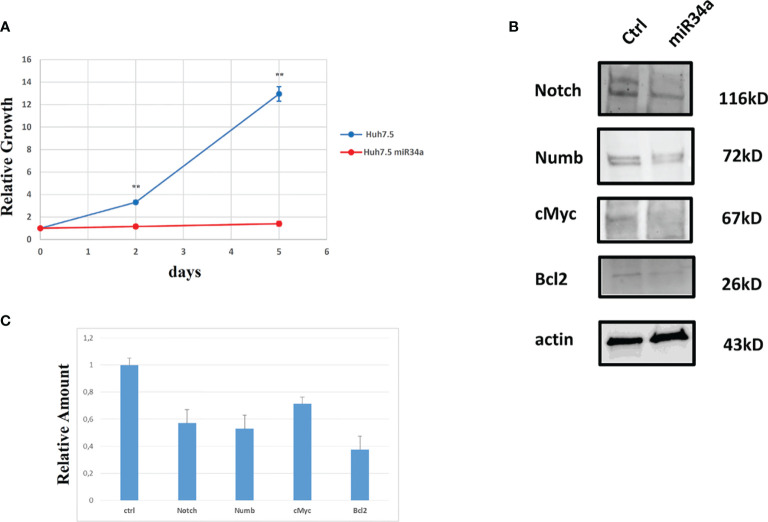
Analysis of Huh7.5 control and overexpressing miR34a: **(A)** Cell growth curve evaluation by CellTiter-Glo (p value **≤0.005); data are representative of experiments performed in triplicate on three different samples. **(B)** Western blot analysis of proteins that have been described as miR34a target and **(C)** protein quantification (p value ≤ 0.05); all experiments have been performed in triplicate.

In view of this remarkable increase, and considering the miR34a role in tumor growth in different tissues/organs (27) and, in particular, the liver (28), we focused on this member of the miR34 family. We evaluated miR34a overexpression effect by using a third-generation lentiviral vector, as previously described (23). Huh7.5-overexpressing miR34a showed a clear morphological change, assessed by bright field microscope observation (**Figure 2**). In particular, it is possible to assess a cellular clear-shape modification after HCV infection, where the infection induces swelling of cells and a density reduction with respect to control cells. Next, we evaluated whether miR34a overexpression in Huh7.5 could interfere with cellular replication, as well as morphology. To this purpose, we performed a cell growth assay. Cell titer growth clearly revealed that miR34a affects HCC cell viability (**Figure 3A**).

**Figure 4:**
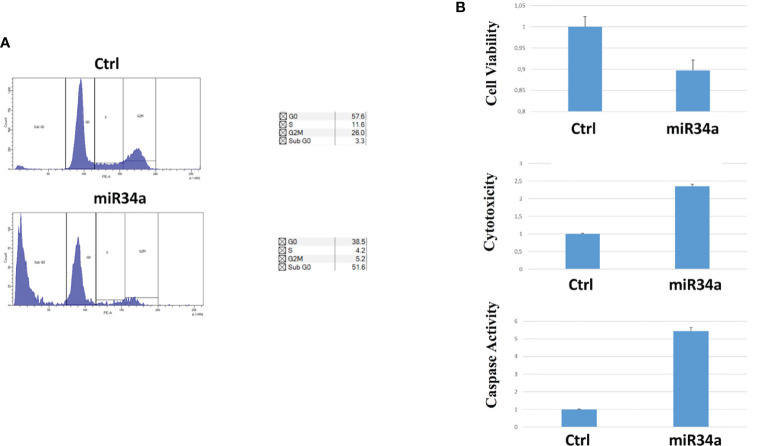
Evaluation of miR34a overexpression effect on Huh7.5: **(A)** propidium iodide cell cycle analysis and **(B)** cell viability, cytotoxicity, and caspase activation (the analysis was carried out in triplicate; p value ≤ 0.05).

To gain insight into the miR34 mechanism of action in Huh7.5, we analyzed the protein expression for some key genes that have been described as targets for this miRNA. In particular, miR34a overexpression in Huh7.5 induces a downregulation for Notch, Numb, cMyc, and Bcl-2 (**Figures 3B, C**). While the first three genes are related to hepatocyte proliferation (24, 29–31) and neoplastic progression (21, 32–34), Bcl-2 reduction may suggest an involvement in apoptotic response (35). We evaluated miR34a-induced apoptosis. In particular, we analyzed the cell cycle profile by propidium iodide (PI) incorporation assay in Huh7.5 overexpressing miR34a (**Figure 4A**). As expected, flow cytometry analysis revealed induction of apoptosis, with an increase of approximately 50% in the sub-G0 population. This result prompted us to evaluate caspase-3/7 activation by ApoTox-Glo Triplex Assay (**Figure 4B**). The analysis confirmed a significant reduction of cell viability, already seen with the CellTiter-Glo analysis, showing a >2-fold increase cell cytotoxicity as a consequence of the miR34 overexpression. Moreover, a >5-fold increase of caspase-3/7 activity together with Bcl2 reduction, compared to the control, indicates the apoptosis induction by the intrinsic pathway.

**Figure 5:**
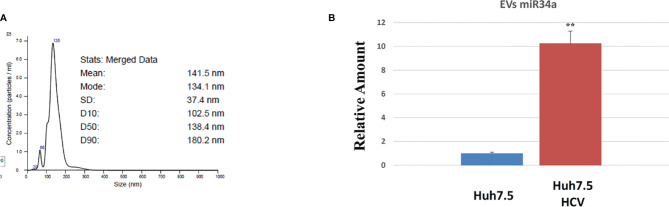
Characterization of Huh7.5 EVs by nanoparticle tracking analysis (NTA) and digital PCR. **(A)** Representative NTA graphs of EV samples obtained after differential ultracentrifugation of secretome, showing size diameter (nm) of particle populations. **(B)** Digital PCR analysis showing the expression of miR34a after HCV infection vs. control The reported data are representative of experiments performed in triplicate on three different samples (**p value ≤ 0.005).

**Figure 6:**
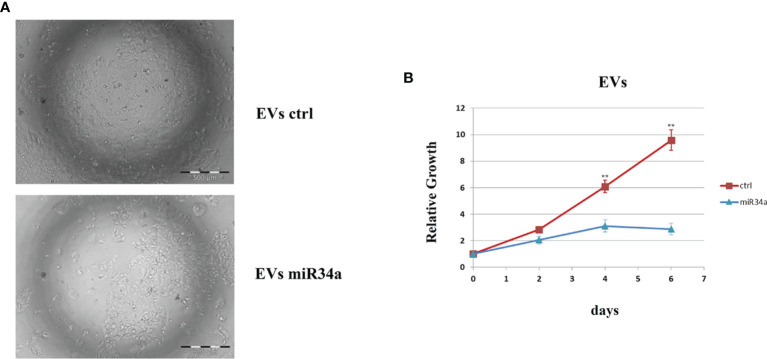
Evaluation of Huh7.5 control and treated with miR34a-loaded exosomes. **(A)** Bright field microscopy and **(B)** cell growth curve by CellTiter-Glo demonstrated that miR34-loaded EVs clearly reduce the cell growth rate (**p value ≤ 0.005). The reported data are representative of experiments performed in triplicate on three different samples.

**Figure 7:**
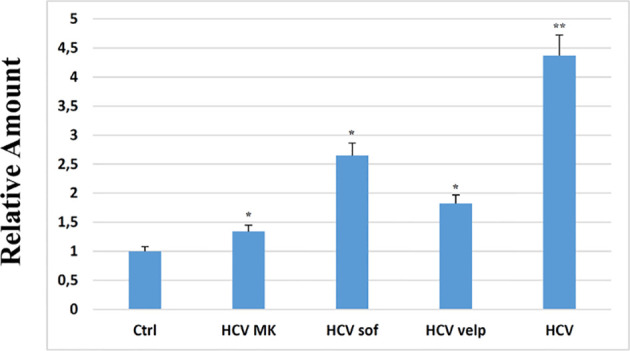
RT-PCR analysis for miR34a in empty Huh7.5 (control, Ctrl), HCV infected, and Huh7.5 treated with sofosbuvir, velpatasvir, and grazoprevir for 3 weeks at a concentration of 10 nM; the absence of virus after treatment was evaluated at 15 days of treatment by immunoassay (data not shown). The reported data are representative of experiments performed in triplicate on three different samples (p value * ≤ 0.05, **≤ 0.005).

All these results demonstrated the miR34a overexpression action in HCC cell lines and suggested that HCV infection induces a cytotoxic/cytostatic effect in target cells. However, since HCV infection is not widespread in all liver cells (36), we evaluated whether miR34 cytostatic/cytotoxic action in infected cells could be transferred to the uninfected cells in a paracrine way. In order to evaluate miR34a expression in the vesicular fraction of Huh7.5, secretome from HCV-infected Huh7.5 was collected, and the released EVs were isolated. EVs purified by differential ultracentrifugation (37) were assessed by Nanoparticle Tracking Analysis (NTA) (**Figure 5A**). Furthermore, a quantitative characterization of miR34a expression in EVs was done through digital PCR analysis. As expected, we found a significant increase of miR34a expression in the secreted fraction (**Figure 5B**), confirming that HCV infection may act not only on miR34a in the infected cells but also on neighboring cells through EV delivery. Consequently, we tested whether miR34a carried by EVs may act similarly to the miRNA overexpressed by vector transduction. We purified the EVs overexpressing miR34a and evaluated their effect on Huh7.5 growth. We observed that miR34a-loaded EVs have a similar effect on cells as its overexpression is induced by lentiviral transduction (**Figure 6**). Microscope evaluation revealed an increase in suffering cells (**Figure 6A**). Similarly, the cell growth curve confirmed that EVs overexpressing miR34 have a cytotoxic effect on Huh7.5 (**Figure 6B**). Finally, we investigated whether DAA treatment could affect miR34 overexpression in HCV-infected Huh7.5. We tested three distinct drugs currently used in HCV treatment: sofosbuvir, velpatasvir, and grazoprevir. By *in vitro* assay, we found a decrease of miR34a expression after 3 weeks of DAA treatment of HCV-Huh7.5 indicating that the HCV effect on miR34 induction was not completely abrogated (**Figure 7**), despite the fact that the virus was totally removed, as assessed by immunoassay (data not shown).

## Discussion

In a previous study, we observed that miR34 was upregulated after ZIKV infection in GSCs (21). Similarly, other authors have described an increase in miR34 expression after HCV infection, particularly in serum of patients with chronic hepatitis C (38). Pairwise, miR34 overexpression has been also correlated with the grade of HCV-dependent cirrhosis, where patients with mild or moderate fibrosis display lower miR34 level expression in the circulating fraction (39). It has been suggested that the *Flaviviridae* infection can enhance the expression of miRNAs in target cells to counteract the viral replication (40). In particular, one study reported that miR34 inhibits viral replication for dengue virus (DENV), West Nile virus (WNV), and Japanese encephalitis virus (JEV), suggesting that miR34 transfection induces the interferon (IFN)-mediated response (40). Another explanation for miR34 expression enhancement is related to p53 induction after HCV infection (**Figure S1** **A**), which can be ascribed to different factors. Indeed, on the one hand, HCV infection can cause double-strand breaks (DSBs) that consequently induce p53 expression (41, 42), and on the other, the HCV core protein stimulates p53-dependent gene expression (43, 44). p53 expression, in turn, acts in a feedback loop with miR34 through Sirt1 (45): p53 induces the miR34 transcription, and miR34 inhibits p53 translation (46, 47). The persistence of miR34a expression observed *in vitro* after viral eradication (**Figure 7**) can be ascribed to the downstream signaling that is not completely abolished. Our work shows that HCV infection induces the expression of miR34a through a mechanism that has not been completely clarified. This produces a cytostatic/cytotoxic effect, leading to tumor suppression (48). Moreover, miR34 induction is not limited to the infected cells but also acts in a paracrine way by EV release from infected cells. This result is supported by the observation that miR34 is clearly enhanced in the serum of infected patients (38, 49).

Likely, our results provide insights on the cytostatic/cytotoxic effect of HCV infection in neoplastic cells (**Figure S1** **B**). On the other hand, this investigation may open a route for a novel therapeutic intervention based on EVs loaded with miR34, or engineered cells, such as MSCs (50), able to release this miRNA by EV delivery (data not shown) for their tumor tropism (51), thus reducing tumor progression. Further studies are needed to confirm this strategy for HCC prevention after HCV eradication.

## Materials and Methods

### Cell Culture, Transfection, and Infection

Huh7.5 cells (a kind gift of Prof. R. Bartenschlager, Heidelberg University, Germany, with the authorization of Apath LLC, NY) (52) were cultivated in DMEM high glucose (Gibco) supplemented with 10% calf serum. For the growth assays, cells were seeded in 6–96-well plates (cell culture treated, Corning, New York, USA). After the indicated time, the cells were collected and lysed with CellTiter-Glo Luminescent 3D Cell Viability Assay reagent (Promega, Mannheim, Germany). Caspase activity was evaluated using the ApoTox Triplex Assay (Promega). Luminescence was analyzed by the Spark Microplate Reader (Tecan, Männedorf, Switzerland).

Huh7.5 transduction was carried out with lentiviral vectors, as previously described (34), with third-generation lentiviral vectors to transduce simultaneously both reporter and miR34a.

Sofosbuvir, velpatasvir, and grazoprevir (Selleckchem, Boston, USA) were resuspended as indicated by the vendor and used at the concentration of 10 nM; the absence of the virus was assessed as described below.

### HCV Core Ag Quantification

Viremia of HCV-infected Huh7.5 culture media was assessed using the two-step chemiluminescent microparticle immunoassay ARCHITECT HCV Ag on the ARCHITECT-i2000R Immunoassay Analyzer (Abbott Diagnostics, Illinois, USA). Briefly, 300 µl of culture supernatants were transferred into 2-ml sample cups prior to loading. Specimens with HCV-Ag concentrations <3 fmol/l were considered non-reactive, while those with HCV-Ag between 3 and 10 fmol/ml (i.e., the “grey-zone”) were retested per manufacturer recommendations.

### Transfection of Huh7.5 Hepatic Cell Line With HCV JFH1 Replicon

Permissive Huh7.5 cells were transfected with HCV RNA by electroporation (3 × 10^6^ cells, 2.5 μg RNA, buffer SE, program CA-138, 4D-Nucleofector, Lonza). Plasmids pFK-Luc-Jc1 and pFK-Venus-Jc1 (GT 2a/2a) were a kind gift of Prof. R. Bartenschlager, Heidelberg University, Germany, with the authorization of Apath LLC, N.Y.) The plasmids were linearized with restriction enzyme Mlu-I and transcribed into HCV RNA with MEGAscript T7 Kit (Ambion), as previously described (3). For pFK-Luc-Jc1, transfection efficiency was monitored with luciferase activity with One-Glo Luciferase Assay System according to the manufacturer’s instructions (Promega). After substrate addition, luminescence was quantified with GloMax 96 Microplate Luminometer (Promega). For pFK-Venus-Jc1, efficiency of transfection was monitored with flow cytometry, as the fluorescent reporter gene Venus emits at a frequency of 527 nm.

### Viral Stock Production

HCV viral stocks were prepared as previously described (3). The HCV strain was 2a. Briefly, culture supernatants of HCV-infected Huh7.5 were clarified of cell debris by low-speed centrifugation (1,000 × g, 4°C, 10 min.), and filtration through a 0.45-μm-pore-size filter. The filtered culture supernatant was buffered with HEPES 20 mM to stabilize pH to about 7.0 and then concentrated by the addition of 1/5 volume of ice-cold 40% PEG-8000/2.5 M NaCl. HCV virus was precipitated at 4°C overnight, followed by centrifugation at 13,000 × g at 4°C for 30 min. The precipitated virus was suspended and stored frozen at −70°C.

### Viral Stock Titration and Immunostaining

Viral stock was titrated as previously described (3). Briefly, 12 × 10^3^/well Huh7.5 were seeded on a 10-mm-diameter glass coverslip placed in a 24-well plate. A 10-fold serially diluted viral stock was added to Huh7.5 cells in a medium that was changed after 6 h. At 72 h postinfection, immunostaining against the HCV core protein was performed. Briefly, cells were washed five times with PBS and fixed with ice-cold 100% methanol at -20°C for 20 min. Cells were washed for five times with PBS; blocked for 1 h with PBS, 5% normal goat serum (BioGenex, Fremont, CA, USA), and 2% BSA; and incubated with mouse anti-HCV Core (clone [C7-50], Abcam, Cambridge, UK) diluted in blocking buffer 1:500 overnight at 4°C in a humidified chamber. Cells were washed with PBS and incubated for 1 h at room temperature with secondary antibody goat anti-mouse IgG-Alexa Fluor-568 (Invitrogen). Stained cells sections were mounted using SlowFade Gold Antifade reagent (Invitrogen) including DAPI for the nuclear counterstaining and stored in the dark. Images were acquired by a confocal microscope (TCS SP5 II, Leica). The number of foci formed at the highest dilution was used to calculate the virus titer, which was expressed as the number of focus-forming units per milliliter of supernatant (FFU/ml). The titers of our JFH1 viral stock were usually in the range of 10^4^ to 10^6^ FFU/ml.

### Propidium Iodide Cell Cycle Analysis

Subconfluent cells were treated as described in the figures. For the analysis, the cells were harvested, washed in PBS, fixed dropwise in 70% cold ethanol (final), incubated 30′ in ice, rewashed in PBS 1% BSA, resuspended in propidium iodide and RNAse (respectively, 50  and 250 µg/ml), and incubated for 60′ RT light protected. DNA content was analyzed with a cytofluorimeter BD FACSCanto II instrument (BD Biosciences, USA).

### RNA Extraction and RTPCR

Total RNA was purified by miRNeasy (Qiagen, Germantown, MD, USA) and reverse-transcribed by TaqMan Universal Mix II (Applied Biosystems, Waltham, MA, USA) using miRNA-specific assay reverse transcription. Semiquantitative PCR was performed with TaqMan-validated assays (Applied Biosystems): miR34a (000426), hsa-miR34b (000427), miR34c (000428), and hsa-miR34c-3p (241009_mat). As reference for cDNA, we chose U6 (#001973) for miRNA. All analyses were carried out in triplicate. Real-time data were collected using Microsoft Excel and analyzed with the following formula: expression level = 2-ΔΔCt method. All experiments were done as independent triplicates and analyzed using standard deviation (SD). The p-value was obtained with the Student’s t-test.

### Immunoblotting

Cells were lysed with a buffer containing 1% Triton X-100, 50 mM HEPES (pH 7.5), 150 mM NaC1, 10% glycerol, 1.5 mM MgCl_2_, 5 mM EGTA, protease inhibitors (4 mM phenyl methylsulfonyl fluoride and 100 mg/ml aprotinin, Sigma-Aldrich), and phosphatase inhibitors (10 mM sodium orthovanadate and 20 mM sodium pyrophosphate, Sigma-Aldrich) and processed. For direct immunoblot analysis, we employed 15–30 μg of total cellular proteins, which were resuspended with 25 μl of loading buffer, boiled for 5 min, and loaded on SDS-PAGE for Western blot (WB). The antibodies for WB were used at the condition suggested by the suppliers: rabbit anti-NOTCH-1 (ab27526, 1/500, Abcam, UK), rabbit anti-Bcl2 (Ab185002, 1/500, Abcam), rabbit anti-human NUMB (ab-14140, 1/1000, Abcam), mouse anti-p53 (ab1101, 1/1000, Abcam), mouse anti-Myc (sc-40, 1/200, Santa Cruz Biotechnology, Texas, USA), and mouse anti-beta-actin (sc-81178, 1/1000, Santa Cruz Biotechnology). The WBs were acquired with the ChemiDoc MP Imaging System (Bio-Rad Laboratories Inc., California, USA), and the corresponding bands were quantified with Image Lab 6.1.0 (Bio-Rad Laboratories, Inc.). The p-value for the relative amount was obtained with the Student’s t-test.

### Extracellular Vesicle Isolation and Characterization

EVs were isolated by differential ultracentrifugation of CM, according to a protocol (53), and analyzed as previously described (37). In brief, cell culture was centrifuged at 300g for 10′ to remove cells, and the supernatant harvested was subsequently centrifuged at 1,800g for 10′ to remove debris, again at 20,000g for 30′, and then at 160,000g for 90′ in the ultracentrifuge (Optima MAX-XP, Beckman Coulter Inc., Irving, TX, USA). All centrifugation steps were performed at 4°C. The pellets were resuspended in phosphate-buffered saline (PBS) without Ca^2+^/Mg^2+^ (Sigma-Aldrich) or subjected to protein or RNA extraction.

Pellet particles resuspended in PBS were analyzed for size and concentration by nanoparticle tracking analysis (NTA) using the NanoSight (NS300, Malvern Instruments, Westborough, MA, USA). Samples were diluted in PBS, 300 μl of samples was loaded into the chamber, and five videos for each sample were recorded. Data analysis was done with the NTA software, and data were presented as mean ± standard deviation (SD) of the five videos.

### Digital PCR

The digital PCR (ddPCR) procedure was performed following/according to the manufacturer’s instructions. The PCR reaction mixture was assembled as follows: ddPCR Supermix for Probes 2 × for probe (no dUTP) (Bio-Rad), 20 × Assay (for miRNA or U6), RNase-free water, and cDNA template 5 μl, in a final volume of 22 μl. Then, a QX200 droplet generator (Bio-Rad) was used to convert 20 μl of each reaction mix into droplets; this produces about 20,000 droplets per sample in about 2.5′ for eight samples. The droplet-partitioned samples were transferred, by pipetting gently, to a 96-well plate, sealed, and processed in a GeneAmp PCR System 9700 Thermal Cycler (Applied Biosystems) under the following cycling protocol: enzyme activation at 95°C for 10′; denaturation at 94°C for 30″ s; annealing/extension at 60°C for 60″ for 40 cycles followed by an infinite 4° hold. The amplified samples were then transferred and read in the FAM and HEX channels using the QX200 reader (Bio-Rad). The experiments were performed using a negative control (no template control, NTC) and a positive control (a sample confirmed positive by RT-PCR with other diagnostic testing). The reactions with less than 10,000 droplets and discordant results were repeated. Data were analyzed using the QuantaSoft™ Software (Bio-Rad). The p-value among the different samples quantification was obtained with the Student’s t-test.

## Data Availability Statement

The raw data supporting the conclusions of this article will be made available by the authors, without undue reservation.

## Author Contributions

EB conducted HCV experiments and performed overall research, data analysis, and manuscript writing. CC performed molecular biology experiments and data analysis. CMC contributed to manuscript editing and discussion. RT contributed to manuscript editing and discussion. PC provided funding support and study design. GI conceived and designed the study, conducted cell biology and biochemistry experiments, and supervised the study and manuscript writing. Data collection and interpretation were performed by all authors. All authors contributed to the article and approved the submitted version.

## Conflict of Interest

The authors declare that the research was conducted in the absence of any commercial or financial relationships that could be construed as a potential conflict of interest.

## Publisher’s Note

All claims expressed in this article are solely those of the authors and do not necessarily represent those of their affiliated organizations, or those of the publisher, the editors and the reviewers. Any product that may be evaluated in this article, or claim that may be made by its manufacturer, is not guaranteed or endorsed by the publisher.

## Supplementary Material

Supplementary Figure 1 Analysis of Huh7.5 control and HCV infected: **(A)** Western blot analysis on/off p53 before and after infection, with a clear increase of protein expression after HCV infection. **(B)** Cell growth curve evaluation by Cell Titer Glo on Huh7.5 control and HCV CI, the viral infection reduces the cell growth rate, the reported data are representative of experiments performed in triplicate on three different samples (p value * ≤ 0.05, **≤ 0.005). **(C)** protein quantification, the analysis was carried out in triplicate (p value ≤ 0.05).

Supplementary Figure 2 Viral expression from the supernatant of Huh7.5 HCV infected and Huh7.5 CI HCV. HCV was quantified as described in material and methods by immunoassay. The analysis has been performed on three different samples (p value ****≤ 0.0001).

Supplementary Figure 3 Digital PCR analysis showing the expression of miR34a after miR34 overexpression vs. control The reported data are representative of experiments performed in triplicate (*p value ≤ 0.05).
