# Supplementary figures and images for: HCV Interplay With Mir34a: Implications in Hepatocellular Carcinoma
Source: Front Oncol. 2022 Jan 19;11:803278. doi: 10.3389/fonc.2021.803278 (PMC8812294; doi:10.3389/fonc.2021.803278)

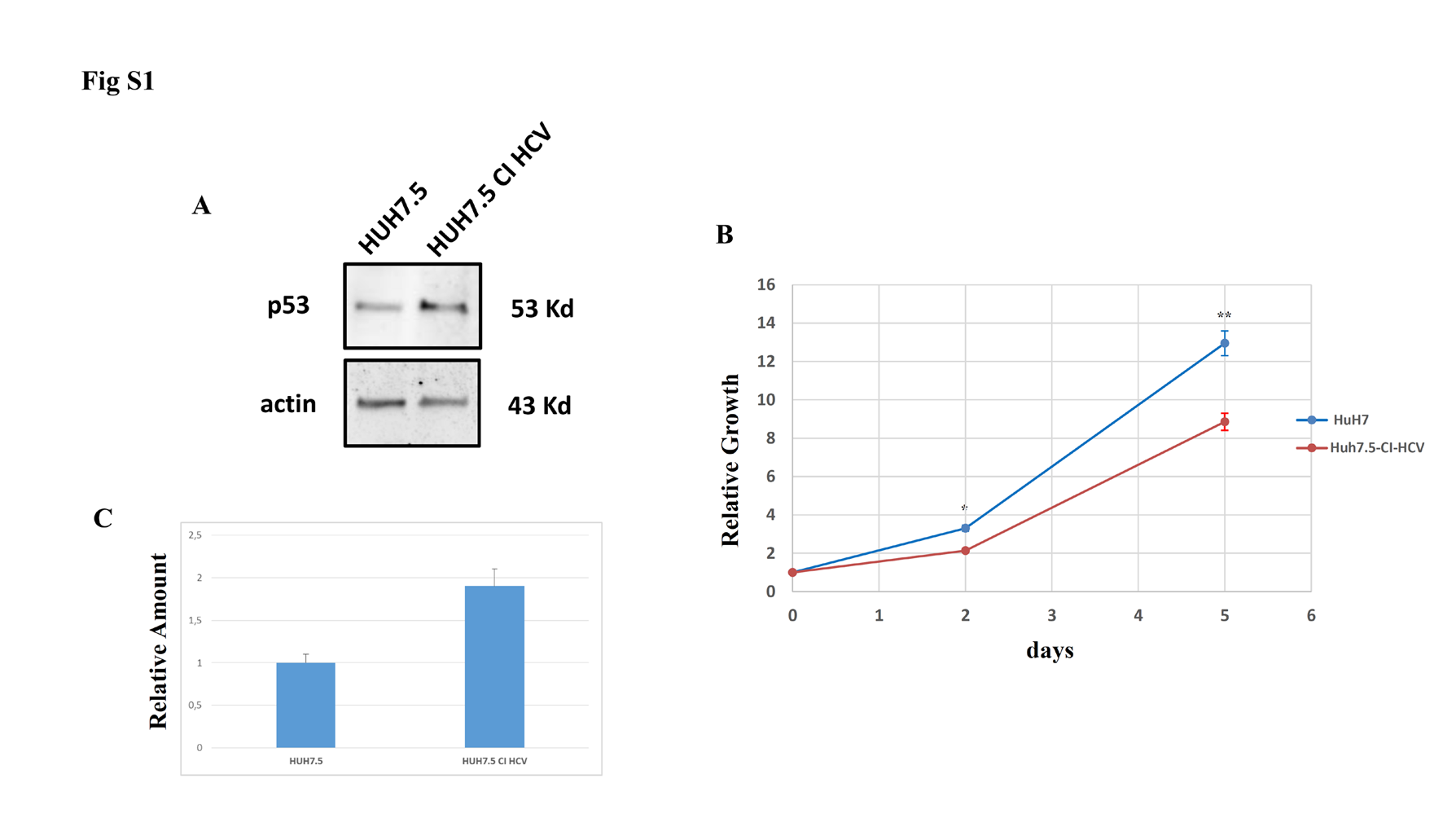

Supplement: Supplementary Figure 1 — Analysis of Huh7.5 control and HCV infected: (A) Western blot analysis on/off p53 before and after infection, with a clear increase of protein expression after HCV infection. (B) Cell growth curve evaluation by Cell Titer Glo on Huh7.5 control and HCV CI, the viral infection reduces the cell growth rate, the reported data are representative of experiments performed in triplicate on three different samples (p value * ≤ 0.05, **≤ 0.005). (C) protein quantification, the analysis was carried out in triplicate (p value ≤ 0.05). [file Image_1.tif]

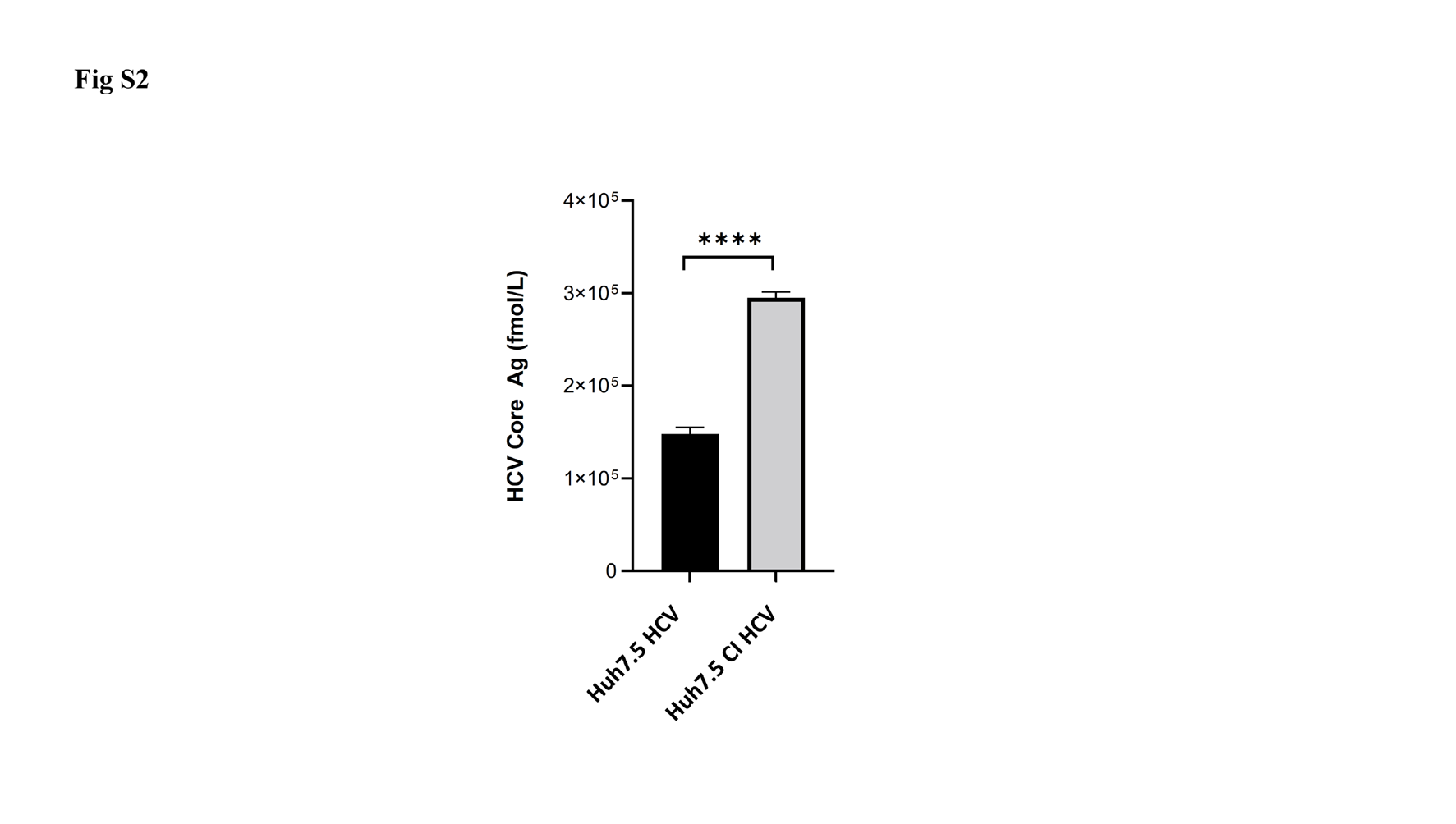

Supplement: Supplementary Figure 2 — Viral expression from the supernatant of Huh7.5 HCV infected and Huh7.5 CI HCV. HCV was quantified as described in material and methods by immunoassay. The analysis has been performed on three different samples (p value ****≤ 0.0001). [file Image_2.tif]

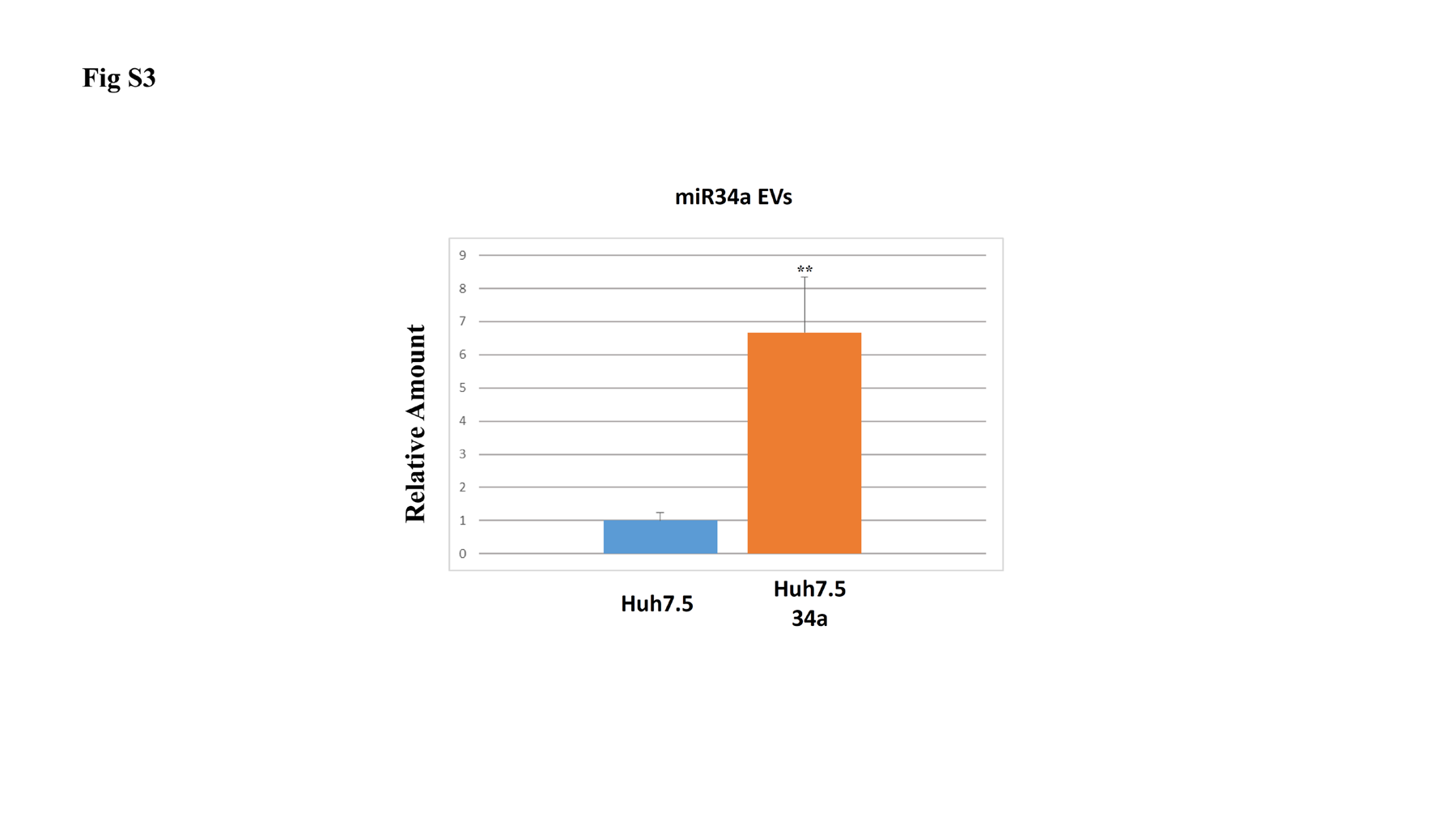

Supplement: Supplementary Figure 3 — Digital PCR analysis showing the expression of miR34a after miR34 overexpression vs. control The reported data are representative of experiments performed in triplicate (*p value ≤ 0.05). [file Image_3.tif]
